# Analysis of Purine Metabolism to Elucidate the Pathogenesis of Acute Kidney Injury in Renal Hypouricemia

**DOI:** 10.3390/biomedicines10071584

**Published:** 2022-07-02

**Authors:** Daisuke Miyamoto, Nana Sato, Koji Nagata, Yukinao Sakai, Hitoshi Sugihara, Yuki Ohashi, Blanka Stiburkova, Ivan Sebesta, Kimiyoshi Ichida, Ken Okamoto

**Affiliations:** 1 Department of Nephrology, Graduate School of Medicine, Nippon Medical School, Tokyo 113-8603, Japan; dm-1010@nms.ac.jp (D.M.); y-sakai@nms.ac.jp (Y.S.); 2 Department of Applied Biological Chemistry, Graduate School of Agricultural and Life Sciences, The University of Tokyo, Tokyo 113-8657, Japan; a-satonana_6775@g.ecc.u-tokyo.ac.jp (N.S.); aknagata@g.ecc.u-tokyo.ac.jp (K.N.); 3 Department of Endocrinology, Diabetes, and Metabolism, Graduate School of Medicine, Nippon Medical School, Tokyo 113-8603, Japan; hitoshi@nms.ac.jp; 4 Department of Pathophysiology, Tokyo University of Pharmacy and Life Sciences, Tokyo 192-0392, Japan; y131047@toyaku.ac.jp (Y.O.); ichida@toyaku.ac.jp (K.I.); 5 Department of Pediatrics and Adolescent Medicine, First Faculty of Medicine, Charles University and General University Hospital, 11000 Prague, Czech Republic; Blanka.Stiburkova@lf1.cuni.cz; 6 Institute of Rheumatology, Institute of Medical Biochemistry and Laboratory Diagnostics, First Faculty of Medicine, Charles University and General University Hospital, 11000 Prague, Czech Republic; isebes@lf1.cuni.cz; 7 Division of Kidney and Hypertension, Jikei University School of Medicine, Tokyo 105-8461, Japan

**Keywords:** hypouricemia, xanthinuria, acute kidney injury

## Abstract

Renal hypouricemia is a disease caused by the dysfunction of renal urate transporters. This disease is known to cause exercise-induced acute kidney injury, but its mechanism has not yet been established. To analyze the mechanism by which hypouricemia causes renal failure, we conducted a semi-ischemic forearm exercise stress test to mimic exercise conditions in five healthy subjects, six patients with renal hypouricemia, and one patient with xanthinuria and analyzed the changes in purine metabolites. The results showed that the subjects with renal hypouricemia had significantly lower blood hypoxanthine levels and increased urinary hypoxanthine excretion after exercise than healthy subjects. Oxidative stress markers did not differ between healthy subjects and hypouricemic subjects before and after exercise, and no effect of uric acid as a radical scavenger was observed. As hypoxanthine is a precursor for adenosine triphosphate (ATP) production via the salvage pathway, loss of hypoxanthine after exercise in patients with renal hypouricemia may cause ATP loss in the renal tubules and consequent tissue damage.

## 1. Introduction

Hypouricemia is defined as a serum uric acid (UA) concentration of less than 2.0 mg/dL [1]. Several diseases cause hypouricemia, but the effect of hypouricemia on the human body is not yet known.

Hypouricemia can be divided into two groups: hypouricemia due to lack of UA synthesis and hypouricemia due to increased renal UA excretion. Hereditary hypouricemia of the former includes hereditary xanthinuria (XU), molybdenum cofactor deficiency, purine nucleoside phosphorylase deficiency, and phosphoribosyl pyrophosphate synthetase deficiency (PRPP) [2]. Hereditary hypouricemia of the latter include renal hypouricemia (RHUC) and Fanconi syndrome [2]. RHUC and XU are the most common diseases that cause hypouricemia with no clinical history and few other subjective symptoms.

In the kidney, UA in the blood is filtered through the glomerulus and then reabsorbed and secreted via UA transporters, such as urate transporter 1 (solute carrier family 22 member 12, urate transporter 1: URAT1) and glucose transporter 9 (solute carrier family 2, member 9 GLUT9) for reabsorption and adenosine triphosphate (ATP)-binding cassette transporter G2 (ABCG2) and organic anion transporter 1 (OAT1) for secretion, which are expressed in proximal tubular epithelial cells [3]. RHUC is caused by loss-of-function mutations in *SLC22A12/URAT1* gene or *SLCA9/GLUT9* gene. RHUC is subdivided into type 1 RHUC (MIM: 220150) caused by loss-of-function mutations in the *URAT1* gene and type 2 RHUC (MIM: 612076) caused by loss-of-function mutations in the *GLUT9* gene [4]. RHUC is a common cause of hypouricemia, which has a prevalence rate of approximately 0.3% in the Japanese population [4,5]. RHUC has significant population specificity: the high incidence of RHUC1 in Japanese and Korean patients reflects the 2.3% allelic frequency of the prevalent dysfunctional variant rs121907892 (p.W258X) [6], with null allele frequency in African/American, Ashkenazi Jewish, South Asia, and European populations (130,978 subjects, ExAc database). The world’s highest frequency of predominant dysfunctional RHUC1 variants was identified in the common European Roma population (1125 subjects): the rs200104135 (p.T467M) variant has a frequency of 5.76%, and the deletion variant p.L415_G417 (no available in public databases) has a frequency of 1.73%. RHUC is asymptomatic, but it can cause clinical complications, such as exercise-induced acute kidney injury (EIAKI) and urinary tract stones [7,8,9]. The hypothesized cause of the renal failure is that RHUC with low UA is affected by ROS and causes renal ischemia due to vasoconstriction. It is also proposed that high concentrations of UA crystallize in the renal tubules might cause renal damage [10], but the evidence for renal failure is not clear.

Uric acid (UA) is known to be an efficient scavenger of reactive oxygen species (ROS) [11,12], and the other hypothesized cause of renal failure is that RHUC with low UA is affected by ROS and causes renal ischemia due to vasoconstriction.

Type 1 XU is a deficiency of xanthine oxidoreductase (XOR), which catalyzes the oxidative hydroxylation of hypoxanthine to xanthine and xanthine to UA, and is an autosomal recessive disease. XU is a relatively rare disease, with more than 100 cases reported to date [13]. XOR is the only enzyme that produces UA, and in its absence, the only physiological source of UA is from food, thus, the blood UA concentration of these patients is typically less than 60 μmol/L. XU type I is asymptomatic in many cases and has a good prognosis, although in some cases, xanthine stones in the urinary tract and oxypurine deposition occur in the muscles and joints [13,14]. However, there are no reports of EIAKI after exercise in xanthinuria.

Despite the loss of a strong antioxidant effect of UA, these hypouricemic cases pass almost asymptomatically, and there are no adverse effects of low UA levels, except for EIAKI. EIAKI can occur in patients with RHUC but with no such report in XU subjects, even though their hypouricemia is similar to that of RHUC subjects. To elucidate the pathophysiology of hypouricemia and the mechanism of EIAKI, we developed a semi-ischemic forearm exercise test, which does not cause renal failure, to reproduce an exercise load condition in hypouricemia subjects [15]. This method allowed us to observe the transition to anaerobic metabolism and the ATP degradation process in the test without causing renal dysfunction. We performed a semi-ischemic forearm exercise test on five healthy subjects, six RHUC subjects and one hereditary type 1 XU subject, and compared differences in purine metabolism to elucidate the mechanism of EIAKI in RHUC. Although only one XU subject could be tested, the data were presented for comparison, since the disease causes hypouricemia by a different mechanism than RHUC.

## 2. Materials and Methods

### 2.1. Semi-Ischemic Forearm Exercise Test

**Figure 1:**
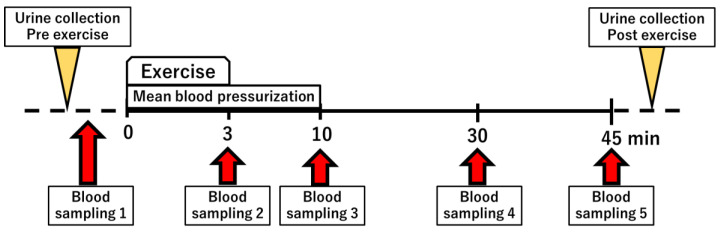
Overview of the semi-ischemic forearm exercise test.

We have previously established a semi-ischemic forearm exercise test that alters purine metabolism [15]. In this study, we performed the exercise test and measured changes in purine metabolites. After 30 min of rest, blood pressure was measured, from which the mean arterial pressure (formula: 2/3 diastolic pressure + 1/3 systolic pressure) was calculated. Subsequently, the initial blood and urine samples were taken, a blood pressure cuff was wrapped around the upper arm, and a 3 min hand grip exercise was started after pressurization at the same pressure as mean blood pressure. A second blood sample was taken at the end of the exercise. After the exercise, pressurization was applied for a further 10 min. The third blood sample was taken at the end of 10 minutes. A fourth and a fifth blood sample were taken 30 and 45 min after the start, respectively (Figure 1). Five healthy subjects, six RHUC subjects (four Japanese and two Czechs), and one XU subject participated in a semi-ischemic forearm exercise test.

This study was conducted with the approval of the Ethics Committee of the University of Tokyo, Jikei University School of Medicine, Tokyo University of Pharmacy and Life Sciences, and Charles University, and informed consent was obtained in advance from all participants.

### 2.2. Analysis of Purine Metabolites

Hypoxanthine (HX), UA, adenosine monophosphate (AMP), adenosine diphosphate (ADP), and adenosine triphosphate (ATP) were measured using high-performance liquid chromatography (HPLC). The separation and quantification of metabolites were performed according to the method of Tani et al. [16] with minor modifications. Blood was treated with 0.91 M perchloric acid to remove proteins. After deproteinization, 3 M potassium carbonate was added to the solution to adjust the pH to 6.6–7.5. Then, 16 μL of the sample and 4 μL of 0.5 M potassium phosphate buffer (pH 5.5) were mixed and injected into the sample loop. Chromatographic analysis was performed using a Shimadzu HPLC instrument (20AD (Shimazu, Kyoto, Japan) equipped with a Shimazu SPD-M20A UV-VIS detector and a SUPELCOSIL LC-18-T column connected to a Supelguard LC-18-T. Purines were separated using a gradient elution system (buffer A: 0.1 M KPB, pH 5.5 containing 4mM tetrabutylammonium hydrogen sulfate; buffer B 30% methanol solution in buffer A) at a flow rate of 1 mL/min. Peaks were detected by measuring absorbance at 254 nm and 295 nm.

ATP is distributed in red blood cells, and the concentration of ATP measured in whole blood varies depends on the excess of red blood cell volume. Therefore, the hematocrit value was used to convert the ATP concentration in whole blood to that in red blood cells. The energy charge (formula: energy charge (EC) = (ATP + 0.5ADP)/(ATP + ADP + AMP)) was also calculated and compared. Lactate and pre- and post-exercise creatinine concentrations were measured by BML Inc. (Tokyo, Japan).

### 2.3. Evaluation of Oxidative Stress Due to Exercise Load

To evaluate oxidative stress due to exercise load, the dicron-reactive oxygen metabolite (d-ROM) level and biological antioxidant potential (BAP) were used. The d-ROM level represents the total level of peroxidized metabolites, and BAP reflects the serum antioxidant capacity [17]. Values for d-ROM and BAP were ascertained using the FREE Carpe Diem (FRAS4; H&D srl, Parma, Italy).

Hydroperoxides, reactive oxygen metabolites (ROMs) present in the blood, generate free radicals in the presence of transition metals, which react with N,N-diethyl-paraphenylendiamine to form a colored derivative. Since color intensity is directly proportional to ROM concentration, peroxidation in the blood can be measured using a spectrophotometer [18]. Levels of d-ROM are expressed in conventional units called U.CARR (Carratelli units), and 1 U.CARR corresponds to 0.8 mg/l H_2_O_2_. Normal levels of d-ROM range between 250 and 300 U.CARR.

BAP measurement is based on the ability of a colored solution containing a source of ferric ions (Fe^3+^) bound to a chromogenic substrate (thiocyanate derivative), to decolor when Fe^3+^ ions are reduced to ferrous ions (Fe^2+^) when exposed to antioxident agents in blood samples [17]. Normal BAP values are greater than 2200 μmol/L.

### 2.4. Adenine Phosphoribosyltransferase and Hypoxanthine Phosphoribosyl Transferase Activity

The activities of adenine phosphoribosyltransferase (APRT) and hypoxanthine phosphoribosyltransferase (HPRT) were determined by HPLC in an erythrocyte lysate according to methods described by Sakuma et al. and Wioleta et al. [19,20]. Erythrocytes were separated by centrifugation at 1500× *g* for 10 min at 4 °C, washed three times with 0.9% NaCl, and frozen at −80 °C. The packed red cells were lysed by freeze–thawing (−80 and 37 °C) twice and diluted with Milli-Q water to adjust hemoglobin (Hb) concentrations to 4 g/dL. Hb concentrations were determined using BML. A reaction mixture containing 250 μL of Reagent A (100 mmol/L Tris-HCl pH 7.4, 12 mmol/L MgCl_2_, 1 mmol/L PRPP, 2 mmol/L HX, 0.2 mmol/L adenine) was incubated for 5 min at 37 °C. Enzyme reactions were initiated by the addition of 25 μL of red cell lysate. After further incubation at 37 °C, 200 μL of the reaction mixture was withdrawn at 5 min and again at 25 min into a tube containing 200 μL of 1.0 mol/L perchloric acid to stop the enzyme reaction. After mixing for approximately 10 s, mixtures were left for 10 min and then centrifuged at 14,000× *g* for 5 min. Supernatants (200 μL) were neutralized (pH 5–7) with 4 M K_2_CO_3_. The samples were centrifuged again under the same conditions as previously described, and 100 μL was used for HPLC analysis to measure the concentrations of inosine monophosphate (IMP) and AMP. The HPLC conditions were the same as those used to measure HX and UA concentration. APRT and HPRT activities were calculated from the increase in AMP and IMP for 20 min and expressed as μmol/min/g Hb.

## 3. Results

### 3.1. Patient Background

We performed the semi-ischemic forearm exercise test in five healthy Japanese subjects, six RHUC subjects (four Czech and two Japanese), and one XU subject. Gene variants (*URAT1*, *GLUT9*, XOR) in the Czech patients (Table 1, cases 6–12) have been published previously [21,22,23,24,25].

Case 6. A 35-year-old man (hereditary type 1 XU): compound heterozygote of two nonsense variants: one base pair deletion in exon 8 (p. P214QfsX4) and one nonsense variant in exon 23 of the *XOR* gene (p. R825X) [21].
Case 7. A 45-year-old man (type 1 RHUC): compound heterozygote of c.1096G>C (p. G366R) in exon 7 and c.1430G>A (p. R477H) in exon 9 of *URAT1*. Variants p.G366R and p.R477H have low urate transport activity, and their combination synergistically loosens the urate transport activity [22].
Case 8. A 49-year-old woman (type 1 RHUC): compound heterozygote of c.1245_1253del (delGGCAGGGCT, p.L415_G417del) in exon 7 c.1400C>T (p. T467M) in exon 9 of *SLC22A12*. The urate transport of both variants was significantly decreased in comparison with the wt (p.L415_G417delP < 0.01, p. T467M *p* < 0.05) [23].
Case 9. A 62-year-old woman (type 1 RHUC): The subject was the older sister of Case 8 and had the same genotype [23].
Case 10. A 58-year-old woman (type 2 RHUC) was heterozygous for the variant c.215G>A (p.G72D) in exon 2 of *URAT1*. The urate transport of the variant was significantly decreased compared to that of wt (*p* < 0.05) [24].
Case 11. A 39-year-old woman (type 1 RHUC): compound heterozygote of c.774G>A (p. W258X;rs121907892) in exon 4 and c.269G>A (p. R90H;rs121907896) in exon 1 of *URAT1*. Both the variants lost the urate transport activity [25].
Case 12. A 42-year-old man (type 1 RHUC): compound heterozygote of c.774G>A (p. W258X;rs121907892) in exon 4 and c.269G>A (p. R90H;rs121907896) in exon 1 of *SLC22A12*. Both variants lost the urate transport activity [25].

### 3.2. Comparison of Lactate and Purine Metabolites in the Exercise Loading Arm and Non-Loading Arm

**Figure 2:**
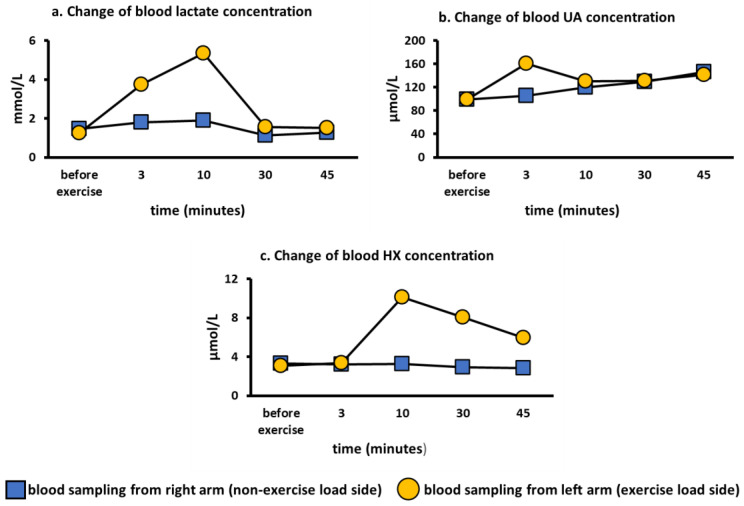
Change of blood lactate, hypoxanthine, and UA concentration on the loading and non-loading sides. HX, hypoxanthine; UA, uric acid. (**a**): Change of blood lactate concentration over time on the loading and non-loading sides. (**b**): Change of blood UA concentration over time on the loading and non-loading sides. (**c**): Change of blood HX concentration over time on the loading and non-loading sides.

To evaluate whether the semi-ischemic forearm exercise test reflected local forearm metabolism or whole-body metabolism, we performed the semi-ischemic forearm exercise test on the left arm in a healthy subject and blood was drawn from left (loaded) and the right (non-loaded) arm. We found that lactate reached a maximum value at 10 min and rapidly decreased after pressure was released (Figure 2a). Blood UA showed a maximum value at 3 min and then showed a gradual downward trend (Figure 2b). Blood HX reached its maximum value at 10 min and then showed a gradual decrease (Figure 2c). These compound level changes upon exercise are consistent with previous reports [15,20,26]. However, none of the analytes were found to increase in the non-loading arm (Figure 2a–c). Therefore, the exercise loading test was found to affect only local metabolism in the forearm.

### 3.3. Comparison of Blood and Urine Analytes in Healthy Subjects and Hypouricemic Subjects

#### 3.3.1. Change of Blood UA Concentration

**Figure 3:**
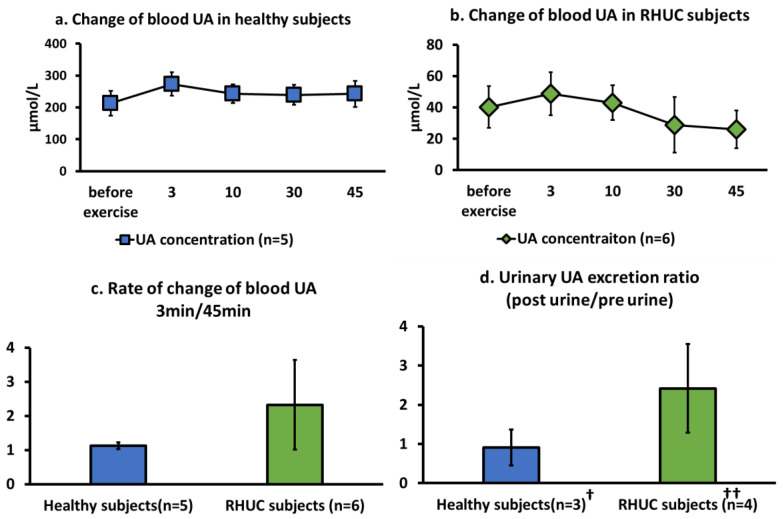
Change of blood UA concentration following semi-ischemic forearm exercise test. (**a**,**b**): Changes of blood UA concentration over time in five healthy subjects and six RHUC subjects. (**c**): The ratio of blood UA concentration at 3 min of maximum value to blood UA concentration at 45 min at the end of exercise load, representing the rate of change of blood UA concentration after exercise load. (**d**): The ratio of urinary UA excretion after exercise to urinary UA excretion per minute before exercise in three healthy subjects and four RHUC subjects, representing the rate of change in urinary UA excretion after exercise. † The three healthy subjects in (**d**) are samples from healthy subject 3, a 24-year-old healthy male, and a 35-year-old healthy man who consented to the study. †† The four RHUC subjects in (**d**) are RHUC 1, 2, 5, and 6. UA, uric acid; RHUC, renal hypouricemia.

RHUC subjects had a significantly very low basal blood UA concentration (t(9) = 8.57, *p* = 0.64 × 10^−7^ < 0.001) due to the increased excretion of UA in the urine, compared to those of healthy subjects (Figure 3a,b). UA concentration in whole blood increased with exercise load, without significant differences, and reached a maximum value at 3 min in both the healthy subjects and the RHUC subjects (Figure 3a,b).

The ratio of the last 45 min of blood UA to the maximum 3 min of blood UA was higher in the RHUC subjects than in the healthy subjects, although the ratio was not significant (Figure 3c).

UA concentration in the blood in healthy individuals is approximately 192 ± 36 μM, when calculated from the serum UA concentration, [27] and the UA concentration in red blood cells, [28] which is consistent with our results. The UA concentration in RHUC was below 120 μM (40.2 ± 13.2 μM), which is one of the diagnostic criteria for RHUC [4]. UA was not detected in the blood of the XU subject.

When the ratio of urinary UA excretion per minute after exercise to that before exercise was compared between normal subjects and RHUC subjects, we observed a tendency for UA excretion to be higher after exercise in the RHUC subjects (Figure 3d).

#### 3.3.2. Change of Blood Xanthine Concentration

**Figure 4:**
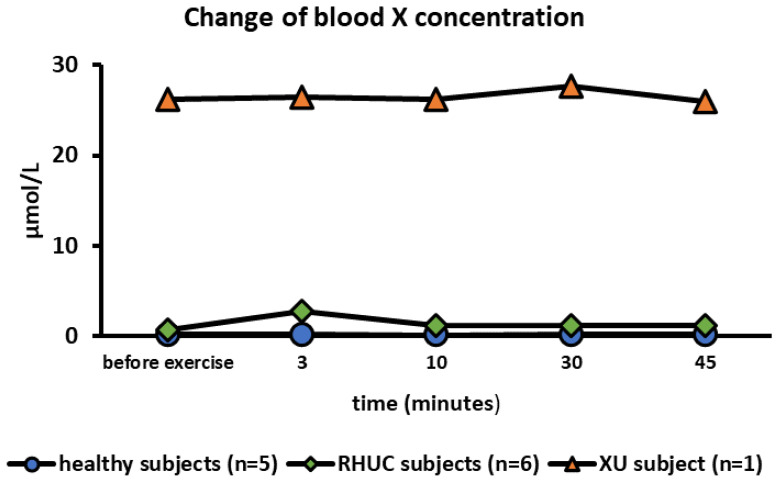
Changes of blood X concentration following semi-ischemic forearm exercise test. X, xanthine.

The mean blood X concentration in healthy subjects at rest was 0.192 μmol/L (range 0–0.681), and in RHUC, 0.753 μmol/L (range 0–1.54), which was almost the same concentration with that in healthy subjects. In the XU subject, however, blood X concentration was extremely high at 26.18 μmol/L at rest. It has been reported that the blood X concentration is high in the XU subject [29]. The blood X concentration hardly changed during exercise (Figure 4).

#### 3.3.3. Change of Blood Hypoxanthine Concentration

**Figure 5:**
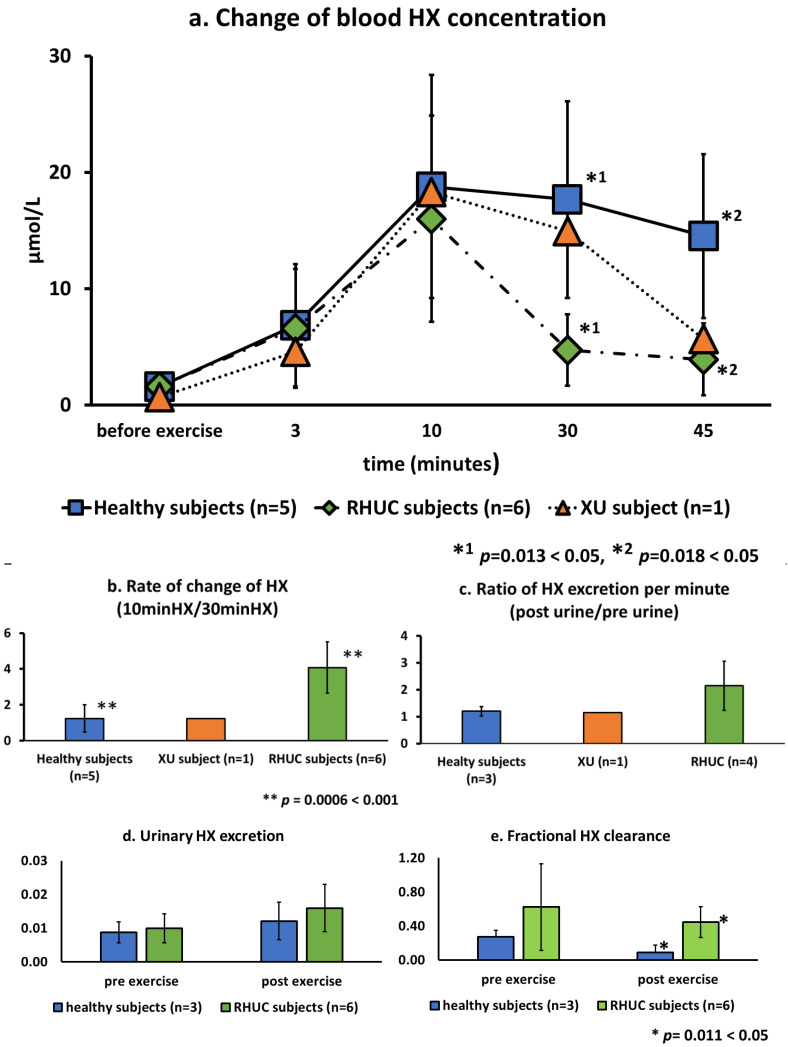
Changes of blood and urine HX concentrations following semi-ischemic forearm exercise test. (**a**): Changes of HX in whole blood of five healthy subjects, one XU subject, and six RHUC subjects after exercise. (**b**): The ratio of blood HX concentration at 10 min of maximum value to 30 min value was taken to show the rate of change of blood HX. (**c**): The ratio of the amount of HX in the urine per minute after exercise to that before exercise. (**d**): Estimated urinary HX excretion by urinary HX/urinary Cre. (**e**). Value of FHX calculated by HX clearance/Cre clearance. HX, hypoxanthine; Cre, creatinine; FHX, fractional HX clearance.

HX concentration in whole blood before exercise were very low, averaging 1.6 μmol/L (range 1.1–2.5) in healthy subjects and 1.6 μmol/L (range 0.2–2.7) in RHUC subjects and the XU subject. However, the HX concentration increased rapidly after exercise, reaching 18.8 μmol/L (range 10.0–32.2) in healthy subjects and 16.0 μmol/L (range 4.3–28.8) in the hypouricemic subjects (Figure 5a). The kinetics of HX after the maximum value showed a gradual decrease in the healthy subjects and the XU subject, while a sharp decrease was observed in the RHUC subjects. The ratio of the maximum blood HX concentration at 10 min to the blood HX concentration at 30 min showed a mean of 1.24 in the normal subjects, 1.23 in the XU subject, and 4.08 in the RHUC subjects, with higher values in the RHUC subjects, and a significant difference in the *t*-test (t(9) = 3.18, *p =* 0.0006 < 0.001) (Figure 5b). In addition, the calculated ratio of urinary HX excretion after and before exercise loading showed a higher tendency in the RHUC subjects (Figure 5c).

To evaluate urinary HX excretion, urinary HX/urinary creatinine (Cre) was calculated, and to evaluate clearance of HX, fractional HX clearance (FHX) (HX clearance/creatinine clearance) was calculated and compared between healthy subjects and the hypouricemic subjects. Urinary HX excretion and FHX before and after exercise were higher in the RHUC group, and FHX after exercise was significantly higher in the RHUC group than in the normal subjects (t(7) = 2.96, *p* = 0.0101 < 0.05) (Figure 5d,e).

#### 3.3.4. Change of Blood ATP Concentration

**Figure 6:**
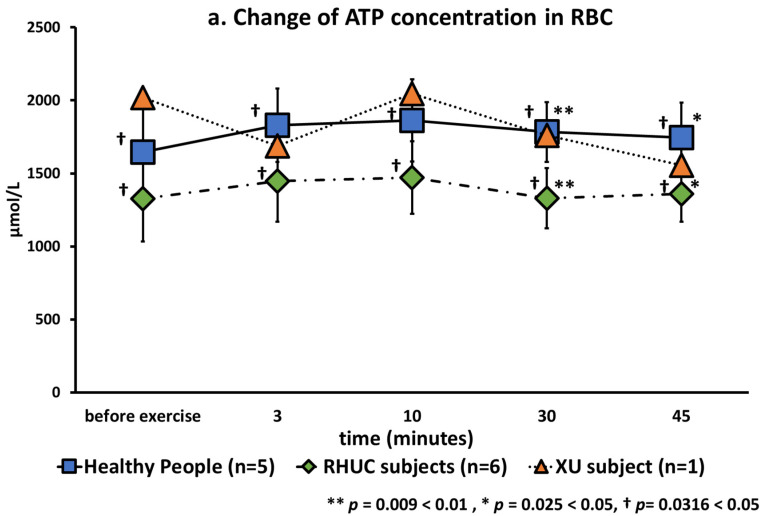

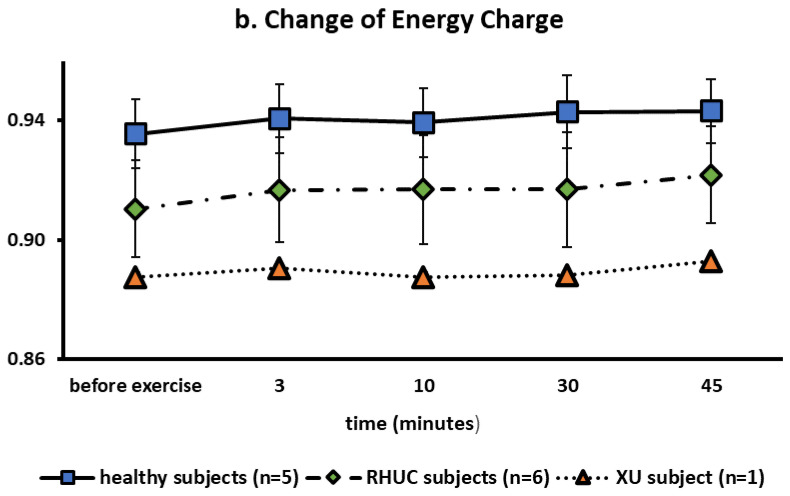
(**a**): Changes of ATP in red blood cells following semi-ischemic forearm exercise test. (**b**): Changes of Energy Charge following semi-ischemic forearm exercise test.

ATP concentrations in blood were stable in healthy and hypouricemic subjects (Figure 6). This is reasonable because red blood cells produce ATP by anaerobic respiration regardless of oxygen concentration.

The ATP concentrations before, 3 min, 10 min, 30 min, and 45 min after exercise were calculated as rows, and those of healthy subjects and RHUC as columns, and analyzed by multivariate analysis of variance (MANOVA). As a result, a significant difference was observed in ATP concentrations between healthy subjects and RHUC subjects (*p* = 0.0316 < 0.05). In conclusion, ATP concentrations were significantly lower in RHUC (Figure 6a).

MANOVA showed significant differences in energy charge of RHUC subjects compared to normal subjects as well as ATP (*p* = 0.042 < 0.05) (Figure 6b).

The mean concentration of ATP in blood was previously reported to be 1729 μmol/L RBC (1622–1741) in healthy subjects [20], which was similar to our results. Regarding energy charge (EC), our results were 0.935 ± 0.011 in healthy subjects, 0.910 ± 0.025 in the RHUC subjects and 0.887 in the XU subject at rest. According to previous reports, EC is between 0.85 and 0.95 in erythrocytes [30], consistent with our results.

#### 3.3.5. APRT and HPRT Activity in RBC

Because changes in blood HX concentrations differed greatly between healthy subjects and RHUC subjects, we analyzed the activities of HPRT, the rate-limiting enzyme of the HX-based salvage pathway, and its analog, APRT in these subjects. There were no differences in APRT and HPRT activities between healthy subjects and the hypouricemic subjects (Table 2), and both were comparable to previously reported values of healthy subjects [19,20]. XOR, another enzyme that uses HX as a substrate, was not measured in this study because of its absence of activity in the red blood cell.

#### 3.3.6. Changes in ROS Markers (d-ROM) and Antioxidant Capacity Markers (BAP)

**Figure 7:**
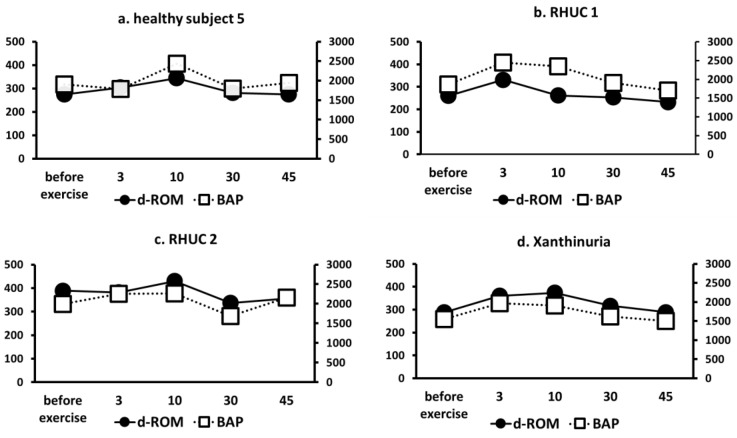
Changes of ROS markers d-ROM and antioxidant capacity marker BAP. (**a**)**:** Healthy subject 5. (**b**): RHUC 1. (**c**): RHUC 2. (**d**): XU. Units on the left vertical axis are U-CARR (d-ROM), units on the right vertical axis are μmol/L (BAP), and the horizontal axis is time (minutes). ROS, reactive oxygen species; d-ROM, dicron-reactive oxygen metabolite; BAP, biological antioxidant potential; RHUC, renal hypouricemia; XU, hereditary xanthinuria.

Using the semi-ischemic forearm exercise test, we compared d-ROMs, a marker of ROS, and BAP, a marker of antioxidant capacity, in one healthy subject, two RHUC subjects, and one XU subject. The results showed that although d-ROMs slightly increased with exercise load in all cases, there were no clear differences between the groups (Figure 7). Furthermore, BAP did not show a significant decrease in antioxidant capacity in response to exercise in the hypouricemic group (Figure 6).

## 4. Discussion

In this study, to elucidate the mechanism of EAKI in RHUC subjects, we performed the previously reported safe semi-ischemic forearm exercise test in healthy subjects, RHUC subjects, and the XU subject, and analyzed the changes in purine compound concentrations in response to ischemic exercise stress.

It is well known that ischemic exercise induces dynamic changes in blood concentrations of many purine compounds, and the patterns of changes and concentrations of these compounds obtained in our experiments were in good agreement with previous reports [15,20,26,31]. Therefore, it is considered that our loading tests are sufficient to discuss metabolic changes.

Phosphorylation of adenine nucleotides occurs by aerobic respiration in mitochondria. When ATP regeneration is insufficient due to ischemic exercise load in the forearm, AMP accumulates. Further hypoxia leads to the degradation of AMPs to maintain the Energy charge [32]. The degraded AMP is quickly metabolized to UA, and UA is released from the tissues. Thus, dynamic changes in purine compounds occur in tissues under hypoxic conditions. In our results, the pattern of variability for some purine compounds was different between healthy subjects and the hypouricemic subjects.

RHUC subjects showed the same increase in UA with exercise load as healthy subjects, but during the recovery period after exercise, blood UA levels rapidly decreased due to rapid transfer into urine. In the XU subject, UA production did not occur even under exercise stress. These findings are consistent with RHUC lacking UA reabsorption and XU lacking protein expression involved in UA production, respectively.

There was a clear difference in the dynamics of HX between healthy subjects and the RHUC subjects. Blood HX concentrations in healthy subjects increased with ischemia and then decreased slowly. From the standpoint of metabolism, elevated levels of HX in the blood reflect increased purine catabolism in tissues caused by AMP degradation during hypoxia. Indeed, Dudzinska et al. observed the changes in blood HX after ischemic exercise using an ergometer and reported that blood HX concentration was maintained in healthy subjects even after exercise, [33] which is consistent with our observation. On the other hand, in RHUC subjects, the blood HX concentration decreased rapidly after exercise. Such difference in HX metabolism between RHUC subjects and healthy subjects has not yet been reported. The rate of decrease in blood HX concentration after exercise in the RHUC subjects was greater than that in healthy subjects and the XU subject, and HX clearance was also greater. In addition, the changes in blood HX concentration and urinary excretion were consistent. These results indicate that the increase in urinary HX excretion in RHUC subjects was the major cause of the rapid decrease in blood HX.

In this study, we observed enhanced urinary HX excretion in the RHUC. Although SLC43A3 has been identified as a transporter involved in HX transport, the details of transporters involved in HX transport in the kidney are not yet known [34]. At least, it has been reported that URAT1 does not directly transport HX [35], indicating that loss of URAT1 indirectly regulates the activity or expression of HX transporters, resulting in increased HX excretion. Further analysis, including the identification of HX transporters, will be necessary in the future.

Erythrocyte ATP levels and EC were significantly lower in RHUC subjects than in healthy subjects. Since RHUC subjects cannot excrete lactate via URAT1, lactate accumulation in erythrocytes is likely to occur, which is expected to inhibit ATP production by anaerobic respiration. However, there are no reports suggesting clinical dysfunction of erythrocytes in RHUC patients. As erythrocytes lack nuclei and mitochondria and do not consume high levels of ATP, even the observed decrease in ATP concentration is not expected to affect their function.

Previously, there has been a report of clinical, biochemical, enzymological, and molecular genetic findings in two XU subjects. They reported that some purine metabolites were significantly changed [21]. In this report, exercise-induced changes in purine metabolites were analyzed. A characteristic finding in the XU subject was that the blood X concentration was high even before the ischemic exercise (Figure 4). In XOR deficiency, HX is not directly metabolized to X. The high blood X concentration in the XU subject indicates that a considerable amount of HX is salvaged to IMP and metabolized to X via guanine. Another interesting feature of the dynamics of X in XU is that, unlike UA and HX, the blood concentration of X is not affected by exercise. This means that the activity of guanase in the body is not high and the rate of X production is saturated.

In the XU subject, XOR, which catalyzes HX catabolism, is defective, resulting in excessive accumulation of HX. However, in our study, the changes in blood HX concentrations in the XU subject at rest and after ischemic exercise were similar to those in healthy subjects. Our results show that the HX clearance at rest in the XU subject is 41.2, which is much higher than 0.27 in healthy subjects and 0.62 in RHUC subjects, and this is similar to previous reports [29]. So, a large amount of the generated blood HX is excreted in urine, which is one of the reasons why blood HX concentrations in the XU subject are not higher than those in healthy subjects.

This section discusses the pathogenesis of EIKAI. RHUC are known to be at risk for EIAKI, although RHUC may be asymptomatic except for hypouricemia [25]. It has been suggested that oxidative stress in RHUC subjects causes vasoconstriction and renal ischemia due to low blood UA, a scavenger of reactive oxygen species [36], or that high levels of UA crystallize in the renal tubules [11]. However, these hypotheses are contradictory and still controversial because EIAKI has not been reported in XU, which causes more severe hypouricemia than RHUC, and renal biopsy results of EIAKI in RHUC show few findings of UA precipitation in the renal tubular lumen or obstructive mechanisms [37].

We investigated whether the development of EIKAI might be due to the ROS scavenging effect of UA. A previous study reported that when exercise loading (treadmill exercise) was performed in healthy subjects and RHUC subjects experiencing EIKAI, BAP decreased after exercise loading in the RHUC subjects, and there was no difference in d-ROM in either group [38]. However, we found that both RHUC subjects and the XU subject had normal antioxidant stress markers and oxidative stress markers before ischemic exercise, and the slight changes caused by ischemic exercise were not significantly different from those in healthy subjects. The difference in the results could be due to the difference in the loading method, and we cannot deny the possibility that the decrease in BAP may be involved in EIAKI. However, our results also suggest that UA does not play a major role in oxidative stress.

Our analysis indicates an association with HX in the pathogenesis of EIKAI. HX is a substrate of the salvage pathway in purine metabolism and can directly make IMP. Our results suggest that in RHUC subjects, the decrease in blood HX, a substrate of the salvage pathway, results in insufficient resynthesis of IMP during exercise. It has been reported that allopurinol, an XOR inhibitor, prevents EIAKI in RHUC [39,40], and is consistent with this hypothesis. Allopurinol can accumulate HX while inhibiting XOR and suppressing UA production. Increased blood HX, a substrate of the salvage pathway, could increase ATP resynthesis and prevent EIAKI in RHUC patients. In the previous studies, the mechanism has been thought to be that allopurinol suppresses the synthesis of UA, thereby reducing the amount of UA filtered by the glomerulus and preventing its deposition in the renal tubules. However, in the pathology of EIAKI in RHUC, there are few reports of UA deposition in the renal tubules [37], and most are not consistent with this finding [10].

It has been reported that HPRT, an enzyme of the salvage pathway, is an enzyme that recycles HX and that its expression does not vary because it is a housekeeping enzyme [41]. Indeed, despite the accumulation of HX, the substrate of HPRT, in the XU subject, the activity of HPRT was the same as in the healthy subject. The activity of APRT, an analogous enzyme, was similarly unchanged. This suggests that in XU patients, an enhanced ATP synthesis from HX occurs homeostatically, and in RHUC subjects, a decrease in blood HX leads directly to a decrease in ATP synthesis via the salvage pathway.

No significant differences were found in the data of Japanese and Czech subjects, except for blood pressure. The average values of blood pressure of the Japanese in our study were 106.6 ± 11.9 for systolic blood pressure (SBP), 70.9 ± 8.8 for diastolic blood pressure (DBP), and 82.7 ± 13.1 for mean arterial pressure (MAP), whereas those of the Czechs were 127.0 ± 4.4 for SBP, 84.2 ± 3.9 for DBP, and 98.0 ± 9.6mmHg for MAP, and Czechs tended to have higher blood pressure. There were no differences in BMI and age. There were no racial differences in exercise-induced changes in purine metabolism. As noted in the Introduction, although the frequency of RHUC is reported to be higher in the Japanese and in the Roma ethnic group, which is also distributed in the Czech Republic [8], there are no reports of differences in the symptoms of RHUC or the frequency of EIAKI.

In conclusion, this study shows that the dynamics of HX could be involved in EIAKI in RHUC subjects. HX is a substrate of the salvage pathway, and the decrease in blood HX in RHUC subjects compared to healthy subjects and the XU subject after ischemic exercise stress indicates that they are unable to resynthesize ATP via IMP and are indicating that they are vulnerable to stress-induced hypoxia. This result is consistent with the report that allopurinol, which inhibits XOR and increases HX, prevents EIAKI in RHUC. Furthermore, it is also consistent with the fact that renal biopsy findings after EIAKI frequently show tubular necrosis. The results of no change in oxidative stress markers and antioxidant capacity markers between the hypouricemic subjects and the healthy subjects also suggest that UA has only a minor effect as a scavenger of ROS in vivo. We are analyzing fluctuations other than purine metabolites in cultured cells and animal models of the disease to understand the pathophysiology of EIAKI in more detail. The results will be published elsewhere.

## Figures and Tables

**Table 1:** Characteristics of the 12 subjects with semi-ischemic forearm exercise test.

Case No.	Age/Sex	Diagnosis	Race	BMI	SBP/DBP/MAP	Notation on the Table and Figure
1	23/F	Healthy subject	Japanese	18.7	92/52/65.3	healthy subject 1
2	23/M	Healthy subject	Japanese	21.2	94/68/76.7	healthy subject 2
3	55/M	Healthy subject	Japanese	30.6	128/82/96.7	healthy subject 3
4	24/M	Healthy subject	Japanese	20.3	108/72/84	healthy subject 4
5	54/M	Healthy subject	Japanese	22.7	118/78/91	healthy subject 5
6	35/M	Xanthinuria	Czech	25.3	130/80/96	XU
7	45/M	Renal hypouricemia	Czech	26.3	133/80/97	RHUC 1
8	49/F	Renal hypouricemia	Czech	46.8	125/90/101	RHUC 2
9	62/F	Renal hypouricemia	Czech	18	127/84/98	RHUC 3
10	58/F	Renal hypouricemia	Czech	21.2	120/87/98	RHUC 4
11	39/F	Renal hypouricemia	Japanese	19.1	102/72/82	RHUC 5
12	42/M	Renal hypouricemia	Japanese	23.4	104/72/83	RHUC 6

SBP; systolic blood pressure, DBP; diastolic blood pressure, MAP; mean arterial pressure, XU; hereditary xanthiuria; RHUC; renal hypouricemia, BMI: body mass index.

**Table 2:** Comparison of purine salvage enzyme activity in RBC. APRT and HPRT activities of healthy subject 5, XU, and three RHUC subjects (RHUC 1, 2, 6) at rest.

	APRT Activity (Unit: μmol/min/g Hb)	HPRT Activity (Unit: μmol/min/g Hb)
healthy subject 5	0.46	2.34
XU	0.67	2.23
RHUC 1	0.69	2.16
RHUC 2	0.65	2.09
RHUC 6	0.51	2.30

APRT, adenine phosphoribosyltransferase; HPRT, hypoxanthine phosphoribosyltransferase, RHUC, renal hypouricemia; XU, hereditary xanthiuria.

## Data Availability

The datasets generated or analyzed during the current study are available from the corresponding author on reasonable request.
